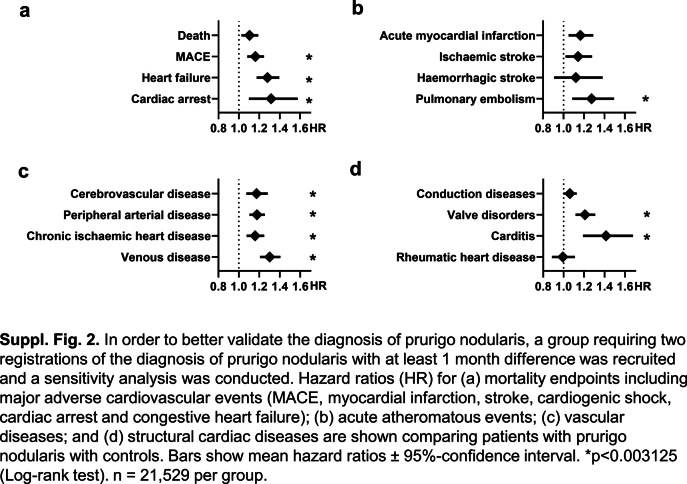# Increased cardiovascular risks and mortality in prurigo nodularis: a global cohort study

**DOI:** 10.1016/j.ebiom.2024.105123

**Published:** 2024-04-16

**Authors:** Henning Olbrich, Khalaf Kridin, Gema Hernández, Henner Zirpel, Christian D. Sadik, Patrick Terheyden, Diamant Thaçi, Ralf J. Ludwig, Katharina Boch

**Affiliations:** a Department of Dermatology, University of Lübeck, Lübeck, Germany; b Unit of Dermatology and Skin Research Laboratory, Galilee Medical Center, Nahariya, Israel; c Lübeck Institute of Experimental Dermatology, University of Lübeck, Lübeck, Germany; d TriNetX, LLC, Cambridge, MA, USA; e Comprehensive Center for Inflammation Medicine, University-Hospital Schleswig-Holstein, Lübeck, Germany; f Department of Dermatology, University of Kiel, Kiel, Germany

**Keywords:** Prurigo nodularis, Cardiovascular disease, Mortality, Myocardial infarction, Stroke, TriNetX

## Abstract

**Background:**

Prurigo nodularis (PN) presents with intensely itchy hard nodules. Despite being limited to the skin, PN was noted to be associated with systemic diseases including diabetes and chronic renal failure. In previous smaller retrospective studies, several cardiac and vascular diseases were found more frequently in patients with PN. However, small cohort sizes, partially discrepant outcomes, missing data, and incomplete risk assessment limit these findings.

**Methods:**

Electronic health records (EHR)s of 64,801 patients (59.44% females) with PN and an equal sized propensity-matched control group were retrieved. In these cohorts, the risks to develop cardiac and vascular diseases and mortality following the diagnosis of PN were determined. Sub-analyses included stratification for sex, ethnicity, and treatments.

**Findings:**

PN was associated with a higher risk for a broad range of acute cardiac events including heart failure and myocardial infarction. For example, the hazard ratio of myocardial infarction was 1.11 (95%-CI: 1.041–1.184, p = 0.0015) following PN diagnosis. Also, all-cause mortality was higher in patients with PN. Further, chronic vascular as well as structural heart diseases, e.g., peripheral arterial disease, chronic ischaemic heart disease and valval disorders were found more frequently following a PN diagnosis. Risks were more pronounced in white and female patients. Having established an increased risk for death and cardiovascular disease, we next addressed if dupilumab that has been recently licenced for use in this indication can modulate these risks. The risk of death but not of any cardiovascular disease was slightly reduced in patients with PN treated with dupilumab as opposed to those treated with systemic therapies other than dupilumab. The study is limited by retrospective data collection and reliance on ICD10-disease classification.

**Interpretation:**

PN is associated with higher mortality and an increased risk for the development of a wide range of cardiac and vascular diseases. Health care professionals should take this into account when managing patients with PN.

**Funding:**

This work was supported by the 10.13039/501100004168University of Lübeck, the 10.13039/501100001659Deutsche Forschungsgemeinschaft and the State of Schleswig-Holstein.

**Research in context**

**Evidence before this study**

Recent cohort studies showed increased risks of several cardiovascular diseases in patients with prurigo nodularis (PN). This included coronary heart disease, congestive heart disease and ultimately also higher mortality. Due to the relatively small sample sizes in the investigated cohorts, the largest including 2409 patients, no further sub-analysis regarding sex and ethnic differences, and no analysis of rarer endpoints was possible thus far. As PN is highly sex- and ethnicity-polarized with significantly more females and more individuals with skin of colour being affected, efficient risk stratification requires diligent individual analysis for otherwise underrepresented groups.

**Added value of this study**

Our study used the large global database of TriNetX to compare cardiovascular outcomes in patients with PN with control individuals. We applied a propensity-score matching strategy in order to exclude other possibly confounding traditional cardiovascular risk factors, other major diseases and demographic differences. We document that PN is associated with higher all-cause mortality, acute myocardial infarction, chronic ischaemic heart disease, peripheral arterial disease and venous diseases. Furthermore, we conducted sub-analyses regarding sex and ethnicity and found that cardiovascular risks were higher in female patients with PN compared with males, and surprisingly, lower in patients of colour with PN compared with whites. In addition, we recruited a cohort of patients with PN treated with dupilumab and compared cardiovascular risks with patients treated with traditional immunosuppressants and antipruritic agents; here, patients treated with dupilumab intriguingly showed lower mortality.

**Implications of all the available evidence**

Patients with PN experience a high comorbidity burden. In particular, we demonstrate cardiovascular risks including acute and chronic vascular outcomes as well as higher all-cause mortality that might arise from a pro-inflammatory cytokine milieu in patients with PN. Females seemed to be more severely affected. Diligent monitoring and awareness of these risks is necessary for patients with PN.

## Introduction

Prurigo nodularis (PN) is a chronic skin condition characterised by intensely pruritic firm hyperkeratotic nodules. The most commonly affected sites include arms, legs, back, and torso, often in a symmetrical distribution.^1^, ^2^, ^3^, ^4^ The quality-of-life burden in PN is often severe and healthcare utilisation is increased.^5^, ^6^, ^7^, ^8^ Overall prevalence is estimated at 72 per 100,000 individuals with a female predominance and higher frequencies in Black and African American individuals.^9^, ^10^, ^11^ PN is associated with a variety of systemic disorders, including chronic kidney and cardiovascular diseases, HIV, chronic hepatitis C infection, chronic obstructive pulmonary disease, but also chronic inflammatory skin diseases like atopic dermatitis, psychiatric comorbidities and neoplasms.^11^, ^12^, ^13^, ^14^, ^15^, ^16^

The precise aetiology of PN remains unknown but is thought to be related to a dysregulated interplay between keratinocytes, immune cells and neurons.^4^ Key mediators include T helper cell (Th)-2 associated factors such as interleukin (IL)-4, -13 and -31 indicating immunological similarities with atopic dermatitis (AD).^17^, ^18^, ^19^ PN is most often diagnosed on a clinical basis; however, histology can be useful to rule out particular differential diagnoses. Blood tests for liver and kidney parameters, glycated haemoglobin (HbA1c) and thyroid function, as well as infectious diseases such as HIV and viral hepatitis can identify underlying systemic diseases.^20^

Treatment of severe forms of prurigo can be challenging and a multimodal approach also considering underlying diseases is advised.^21^ Topical corticosteroids and oral antihistamines are commonly prescribed. Second line options include traditional immunsuppressants and immunomodulators as well as UV-therapy. More recently, dupilumab, an anti-IL-4 and -13 inhibitory monoclonal antibody, has shown efficacy in clearing treatment-resistant PN lesions and was approved for PN-treatment in 2022.^22^ Further therapeutic options for PN that are currently evaluated in clinical trials include the monoclonal antibody vixarelimab targeting the receptor for IL-31 and oncostatin M,^23^ and the anti-IL-31 monoclonal antibody nemolizumab.^24^

In previous smaller retrospective studies, several cardiac and vascular diseases were found to be more frequent in patients with PN compared to patients with AD or psoriasis including hypertension and ischaemic heart disease. These studies, however, did not elucidate a temporal relationship between PN and cardiovascular diseases and low case numbers prevented the detection of rare outcomes and efficient risk stratification based on demographics and comorbidities.^13^ In addition, outcomes after different treatment regimens were not analysed previously. These insights are, however, crucial for efficient secondary prophylaxis and screening of patients with PN.

We thus conducted a large global longitudinal study using electronic health reports (EHR)s provided by the Global Collaborative Network of TriNetX. The database comprises EHRs from 113 health care operations (HCO)s worldwide containing data on diagnoses in the form of ICD-10 codes, medications and medical procedures (see^25^ for an extensive description of the database). The Global Collaborative Network was chosen as it has large numbers of EHRs and a high degree of data coverage and completeness among the networks provided by TriNetX. It includes HCOs from 30 countries in North- and South-America, Europe, the Middle East, Africa, and the Asia–Pacific regions and uploads of EHRs are conducted daily by the individual HCOs. Several strategies for harmonisation and integration of various data sources are implemented in the database.

We evaluated mortality risks as well as the risks of developing defined cardiac and vascular diseases after diagnosis of PN. We compared risks in the patient group with a control cohort stringently matched by traditional Framingham cardiovascular risk factors as well as other major comorbidities. Further, we stratified both cohorts and conducted sub-analyses with respect to ethnic background and sex. Finally, we compared patients with PN treated with standard first- and second-line therapies to patients treated with the recently approved drug dupilumab.

## Methods

### Study design and database

A global population-based retrospective cohort study with propensity-score matching was performed following previously published protocols.^26^, ^27^, ^28^ In detail, we used the Global Collaborative Network of TriNetX to identify EHRs of patients with PN and performed retrospective cohort studies to determine the risk for 15 cardiovascular endpoints and mortality. Endpoints were defined by clinically relevant combinations of ICD-10 codes selected prior to data collection in accordance with recent similar studies.^29^^,^^30^ The ICD-10 codes used are represented in Table 1.

**Table 1:** Definitions of clinical endpoints investigated in the study.

	Outcome	ICD-10 codes
Mortality	All-cause death	–
	MACE	I21, I22, I63, I61, I62, I50, I46, R57.0
	Heart failure	I50
	Cardiac arrest	I46, R57.0
Acute events	Acute myocardial infarction	I21, I22, I25.2, I23
	Ischaemic stroke	I63, I69.3
	Haemorrhagic stroke	I60, I61, I62, I69.0,1,2
	Pulmonary embolism	I26
Vascular disease	Cerebrovascular diseases	I67, I65, I66, I68.0
	Peripheral arterial disease	I96, I73, I70, I77, I71, I74, I78, I79, I75
	Chronic ischaemic heart disease	I20, I24, I25 (except for I25.2), Z95.1
	Thrombotic venous disease	I87.0, I80, I82, I83.0
Structural disease	Valve disorders	I35, I34, I36, I37, Z95.2, Z95.3, Z95.4
	Rheumatic heart disease	I01, I02, I05-09
	Carditis	I51.4, I38, I33, I30, I41, I32, I40, I39, I43, J10.82, B33.2
	Conduction diseases	I44, I45, I47, I48, I49, Z95.0

MACE, major adverse cardiac events.

### Ethics statement

The data reviewed is a secondary analysis of existing data, does not involve intervention or interaction with human subjects, and is de-identified per the de-identification standard defined in Section §164.514(a) of the HIPAA Privacy Rule. The process by which the data is de-identified is attested to through a formal determination by a qualified expert as defined in Section §164.514(b) (1) of the HIPAA Privacy Rule. This formal determination by a qualified expert refreshed on December 2020. Thus, our study did not require Institutional Review Board approval. The study was reported according to the STROBE guidelines.

### Study population and definition of eligible patients

**Supplementary Fig. S1:**
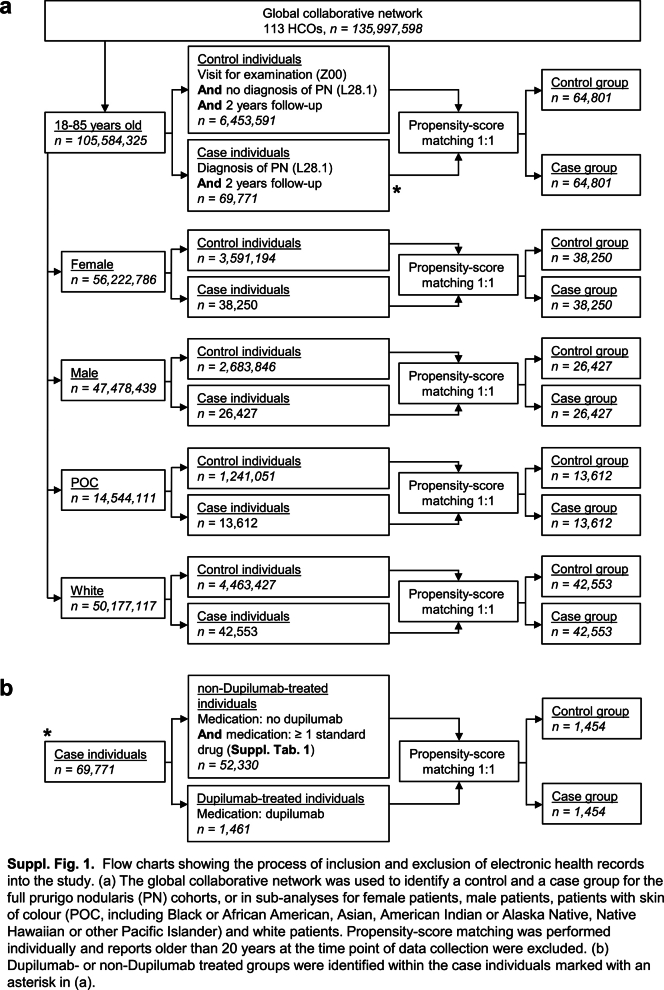


Data for this study was collected in August 2023 and January 2024 from the Global Collaborative Network of TriNetX. The Global Collaborative Network was chosen as it includes the largest number of EHRs among the different networks provided by TriNetX. The network provides access to EHRs from 130 million patients in 113 healthcare organizations (HCO)s worldwide at the time of data collection. We included patients aged 18–85 years with any follow-up visit after at least 2 years to ensure availability of subsequent records after the diagnosis of PN. Patients that met the index event more than 20 years ago were excluded. We enroled 64,801 patients with PN (ICD10: L28.1), a control group with 6,453,591 patients was identified by “Encounter for general examination without complaint, suspected or reported diagnosis” (ICD10: Z00) without the diagnosis of PN at the first or a required ≥2-year follow-up visit. For sex stratification, the groups were split in EHRs reporting either female or male. An ethnicity-specific analysis was conducted by identifying EHRs reporting patients with skin of colour (including patients identifying as Black or African American, Asian, American Indian or Alaska Native, Native Hawaiian or other Pacific Islander), or EHRs reporting White patients. In addition, the patient group was stratified into a dupilumab-treated group and a group that received standard treatment according to the guidelines, but no dupilumab (including a variety of glucocorticosteroids, antihistamines, calcineurin inhibitors, tricyclic antidepressants, gabapentinoids, opioid receptor-antagonists, capsaicin, mycophenolate, ciclosporine or azathioprine, see Supplementary Table S1). A sample-size flow chart is provided in Supplementary Fig. S1. For a sensitivity analysis, we identified a cohort of patients with PN and a subsequent additional diagnosis of PN at least 1 month after the initial diagnosis, as well as a ≥2-year follow-up visit; this is to ensure validity of the diagnosis. A total of 88–100 HCOs provided EHRs matching our inclusion criteria per group, however, data for the ethnicity-specific sub-analysis were only reported by 52–71 HCOs. Baseline characteristics for additional demographics and diagnoses are shown in Table 2 for the full cohort and in Supplementary Table S2 for the dupilumab-treatment subanalysis.

**Table 2:** Baseline characteristics before and after propensity-score matching.

Characteristic	Before matching						After matching					
	Patients		Controls		p-value	SMD	Patients		Controls		p-value	SMD
Age at Index (years, ±SD)	54.81	±15.28	44.64	±19.49	<0.001	0.581	54.81	±15.28	54.92	±15.29	0.217	0.007
ICD-10	n =	%	n =	%			n =	%	n =	%		
Total	64,801	100.00%	6,343,415	100.00%			64,801	100.00%	64,801	100.00%		
Female^a^	38,515	59.44%	3,681,653	58.04%	<0.001	0.028	38,515	59.44%	38,566	59.52%	0.773	0.002
Male	26,275	40.55%	2,651,428	41.80%	<0.001	0.025	26,275	40.55%	26,146	40.35%	0.465	0.004
Unknown Sex	11	0.02%	10,334	0.16%	<0.001	0.049	11	0.02%	89	0.14%	<0.001	0.043
White^a^	41,084	63.40%	4,241,210	66.86%	<0.001	0.073	41,084	63.40%	41,130	63.47%	0.791	0.001
Black or African American	10,608	16.37%	970,716	15.30%	<0.001	0.029	10,608	16.37%	11,804	18.22%	<0.001	0.049
Asian	2418	3.73%	188,517	2.97%	<0.001	0.042	2418	3.73%	2137	3.30%	<0.001	0.024
Unknown Race	10,332	15.94%	910,857	14.36%	<0.001	0.044	10,332	15.94%	9322	14.39%	<0.001	0.043
Not Hispanic or Latino^a^	43,809	67.61%	4,244,815	66.92%	<0.001	0.015	43,809	67.61%	43,823	67.63%	0.934	<0.001
Hispanic or Latino	4181	6.45%	575,094	9.07%	<0.001	0.098	4181	6.45%	4657	7.19%	<0.001	0.029
Unknown Ethnicity	16,811	25.94%	1,523,506	24.02%	<0.001	0.044	16,811	25.94%	16,321	25.19%	0.002	0.017
Family history of ischemic heart disease and other diseases of the circulatory system^a^												
Z82.4	2917	4.50%	275,550	4.34%	0.050	0.008	2917	4.50%	2885	4.45%	0.667	0.002
Overweight, obesity and other hyperalimentation^a^												
E65-E68	16,938	26.14%	1,151,774	18.16%	<0.001	0.193	16,938	26.14%	16,920	26.11%	0.909	0.001
Nicotine dependence^a^												
F17	10,369	16.00%	593,055	9.35%	<0.001	0.201	10,369	16.00%	10,257	15.83%	0.395	0.005
Essential (primary) hypertension^a^												
I10	28,656	44.22%	1,875,841	29.57%	<0.001	0.307	28,656	44.22%	28,619	44.16%	0.836	0.001
Diabetes mellitus^a^												
E08-E13	14,487	22.36%	741,779	11.69%	<0.001	0.287	14,487	22.36%	14,404	22.23%	0.580	0.003
Disorders of lipoprotein metabolism and other lipidemias^a^												
E78	27,270	42.08%	2,005,922	31.62%	<0.001	0.218	27,270	42.08%	27,237	42.03%	0.853	0.001
Neoplasms^a^												
C00-D49	37,385	57.69%	1,438,451	22.68%	<0.001	0.765	37,385	57.69%	37,389	57.70%	0.982	<0.001
Chronic kidney disease^a^												
N18	6272	9.68%	245,189	3.87%	<0.001	0.233	6272	9.68%	6032	9.31%	0.023	0.013
Chronic lower respiratory diseases^a^												
J40-J47	17,134	26.44%	1,027,458	16.20%	<0.001	0.252	17,134	26.44%	17,013	26.25%	0.445	0.004
Noninfective enteritis (incl. Crohn's and Colitis ulcerosa)												
K50–K52	10,176	15.70%	436,985	6.89%	<0.001	0.281	10,176	15.70%	6147	9.49%	<0.001	0.188
Gastro-oesophageal reflux disease												
K21	18,849	29.09%	1,086,003	17.12%	<0.001	0.287	18,849	29.09%	16,910	26.10%	<0.001	0.067
Gastritis and duodenitis												
K29	5516	8.51%	229,234	3.61%	<0.001	0.206	5516	8.51%	3972	6.13%	<0.001	0.092
Other rheumatoid arthritis												
M06	2322	3.58%	87,265	1.38%	<0.001	0.142	2322	3.58%	1587	2.45%	<0.001	0.066
Systemic lupus erythematosus												
M32	995	1.54%	31,352	0.49%	<0.001	0.104	995	1.54%	545	0.84%	<0.001	0.064
Atopic dermatitis												
L20	6417	9.90%	88,851	1.40%	<0.001	0.375	6417	9.90%	858	1.32%	<0.001	0.379
Psoriasis												
L40	4543	7.01%	87,868	1.39%	<0.001	0.283	4543	7.01%	1293	2.00%	<0.001	0.244
Affective disorders												
F30–F39	19,252	29.71%	1,082,936	17.07%	<0.001	0.302	19,252	29.71%	14,536	22.43%	<0.001	0.166
Depressive episode												
F32	16,549	25.54%	888,062	14.00%	<0.001	0.293	16,549	25.54%	12,302	18.98%	<0.001	0.158
Anxiety disorders												
F40–F48	19,604	30.25%	1,321,039	20.83%	<0.001	0.217	19,604	30.25%	16,371	25.26%	<0.001	0.112
Phobic anxiety disorders												
F40	1183	1.83%	49,699	0.78%	<0.001	0.092	1183	1.83%	700	1.08%	<0.001	0.062
Schizophrenia												
F20	704	1.09%	37,215	0.59%	<0.001	0.055	704	1.09%	535	0.83%	<0.001	0.027
Alcohol related disorders												
F10	3659	5.65%	172,046	2.71%	<0.001	0.147	3659	5.65%	2563	3.96%	<0.001	0.079
Cannabis related disorders												
F12	1250	1.93%	79,008	1.25%	<0.001	0.055	1250	1.93%	1077	1.66%	<0.001	0.020
Opioid related disorders												
F11	1586	2.45%	69,370	1.09%	<0.001	0.103	1586	2.45%	1071	1.65%	<0.001	0.056

SD, standard deviation; SMD, standardised mean difference.

a Covariates included in the propensity-score matching model; uncorrected p-values are shown (t-test).

### Covariates

Propensity-score matching (PSM) is performed to match cohorts in observational studies in order to equalise the distribution of potential confounding covariates in case and control groups. Several previous studies have used this approach in order to limit possible bias, and PSM might be advantageous compared with traditional covariate adjustment.^31^ Here, PSM was performed for each sub-analysis by establishing a covariate matrix including known cardiovascular risk factors according to the Framingham risk score for cardiovascular disease,^32^ relevant concomitant diagnoses, and demographic information. Of note, demographic data regarding ethnicity and its classification used by HCOs is not based on biological or genetic differences but rather serves as a surrogate parameter for several socioeconomic factors. A total of 13 covariates were included: age at index, female sex, ethnicity, family history of ischaemic heart disease and other diseases of the circulatory system (ICD10: Z82.4), nicotine dependence (ICD10: F17), obesity (ICD10: E65-68), disorders of the lipoprotein metabolism (ICD10: E78), essential hypertension (ICD10: I10), all types of diabetes mellitus (ICD10: E08-E13), chronic lower respiratory diseases (ICD10: J40-J4A), chronic kidney disease (ICD10: N18) and all types of neoplasm (ICD10: C00-D49). The matrix row order was randomised after data retrieval. A propensity-score for each patient was generated by logistic regression and matching was performed 1:1 using the greedy nearest neighbour approach with a calliper distance of 0.1 using the software package scikit-learn in Python.^33^ Baseline characteristics were re-evaluated and reported after matching, differences were compared by t-test for continuous and z-test for binary or categorical variables.

### Statistical analysis

The statistical analysis was performed using the Survival package v3.2–3 in R (R Foundation for Statistical Computing, Vienna, Austria) and validated by comparison with the outputs of SAS version 9.4 (SAS, Cary, NC); graphs were created using GraphPad Prism (GraphPad Software Inc., Boston, MA). The index event was set as the diagnosis of PN, or the reported healthcare encounter in the control group, respectively. Outcomes at up to ten years after the index event were considered in the analysis. Outcomes prior to the index events were excluded. We analysed cardiac and vascular composite endpoints in the group with PN as well as all-cause death. Relative risks and risk difference (RD) were calculated. Survival analyses were performed using the Kaplan–Meier method (KM). The proportionality assumption was tested by the cox.zph() function in R's Survival package and was true for all comparisons. Further, a competing risk analysis was conducted using Aalen–Johansen plots; here we found no overestimation of possibly competing risks comparing Aalen Johansen and 1-KM estimators. KM-curves were compared using the Log-rank test. Using the Bonferroni correction, we adjusted an initial α = 0.05 to control for the bias imposed by multiple testing for 16 outcomes (adjusted α = 0.05/16 = 0.003125); thus, p-values of less than 0.003125 were considered significant. A univariate Cox proportional hazards regression was used to express hazard ratios (HR)s with 95%-confidence intervals (CI)s.

### Role of funders

The funders had no role in the study design, data collection, data analyses, interpretation, or writing of the report.

## Results

### Cohort description and patient characteristics

Prior to propensity-matching, we identified two cohorts of 64,801 patients with PN (from 96 HCOs) and 6,453,591 control patients without PN (from 100 HCOs). The median follow-up period was 1597 days for the group with PN and 1171 days for the control group. Table 2 shows relevant baseline variates: patients with PN were slightly older with a mean age at index at 54.81 years (SD ± 15.28) for PN and 44.64 (SD ± 19.49) for controls (p < 0.0001). A slight female predominance was noted (59.44% for PN vs. 58.04% for controls; p < 0.0001). Further differences in demographics were minor, but the group with PN included slightly more patients of Black or African American descent (16.37% vs. 15.3%; p < 0.0001) and less patients of Hispanic or Latino ethnicity (6.45% vs. 9.07%; p < 0.0001). Cardiovascular risk factors were more frequent in the group with PN, including nicotine dependence (16.00% vs. 9.35%; p < 0.0001), obesity (26.14% vs. 18.16%; p < 0.0001), hypertension (44.22% vs. 29.57%; p < 0.0001) and diabetes mellitus (22.36% vs. 11.69%; p < 0.0001). Other major concomitant diseases were more frequent in patients with PN, including neoplasms (57.69% vs. 22.68%; p < 0.0001) as well as neuropsychiatric diseases, e.g., a prior depressive episode (25.54% vs. 14.00%; p < 0.0001), but also anxiety, alcohol, cannabis and opioid abuse. To allow for better comparability, a stringent matching model was employed and cases and controls were matched 1:1 for age, female sex, ethnicity, relevant cardiovascular risk factors and other major diagnoses. Matching turned out statistically effective for all selected diagnoses, female sex and White descent, while Black and African American patients were slightly underrepresented in the group with PN (Table 2).

### Major cardiovascular events and mortality

**Fig. 1:**
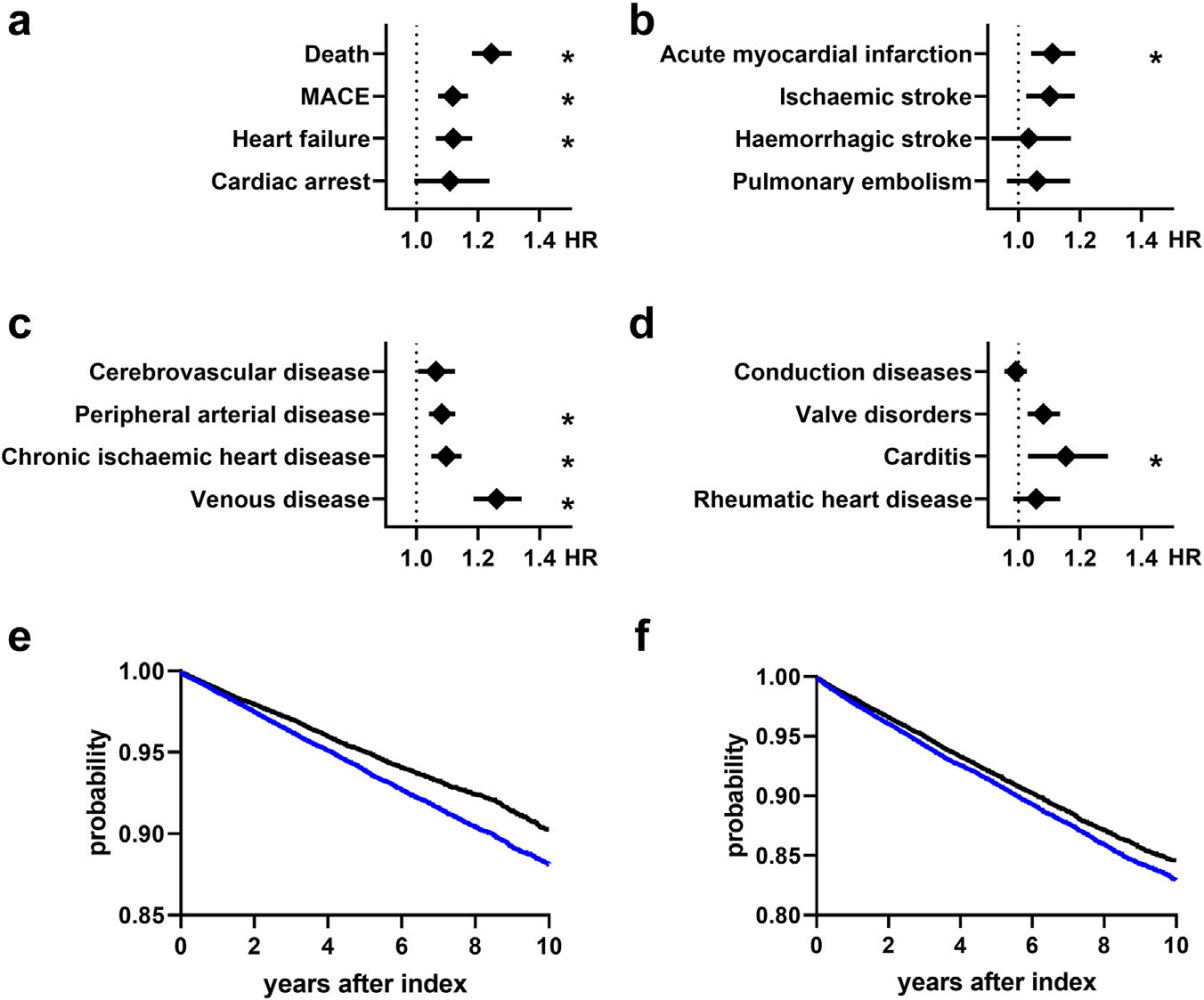
Hazard ratios (HR) for (a) mortality endpoints; major adverse cardiovascular events (MACE) include myocardial infarction, stroke, cardiogenic shock, cardiac arrest and congestive heart failure, (b) acute atheromatous events, (c) vascular diseases and (d) structural cardiac diseases are shown comparing patients with prurigo nodularis with controls. Bars show mean hazard ratios ±95%-confidence interval. ∗p < 0.003125 (Log-rank test). (e) Kaplan-Meyer curves showing survival probabilities regarding mortality and MACE (f) over time for patients with PN (blue) and controls (black). n = 64,801 per group.

In order to estimate mortality related to cardiovascular diseases, we constructed a composite endpoint reflecting major adverse cardiovascular events (MACE) with acute cerebral and myocardial infarction, heart failure, ventricular arrhythmia and sudden cardiac death, that demonstrated an increased risk in patients with PN (HR 1.117; 95%-CI 1.07–1.167; p < 0.0001, see Fig. 1a and f, Table 3). The median lag time between the diagnosis of PN and MACE was 1001 days. Next, we analysed heart failure individually and found higher risks in patients with PN (HR 1.062; p < 0.0001). The rare event of sudden cardiac arrest did not differ significantly. Lastly, all-cause mortality was analysed and showed a higher risk in patients with PN (HR 1.1243; CI 1.18–1.309; p < 0.0001, see Fig. 1e). All endpoints were analysed within a ten year period after diagnosis of PN.

**Table 3:** Cardiovascular outcomes after diagnosis of Prurigo nodularis (PN) showing risk difference (RD) and hazard ratios (HR) with 95%-confidence intervals (CI).

Outcome	PN-patients			Controls			Risk difference (RD)		Hazard ratio (HR)		p-value (log-rank)
	patients in cohort	patients with outcome	risk (%)	patients in cohort	patients with outcome	risk (%)	RD (%)	95%-CI	HR	95%-CI	
Death	64,315	3579	5.565%	64,439	2435	3.779%	1.786%	(1.556%, 2.016%)	1.243	(1.18, 1.309)	<0.0001
MACE	57,394	4666	8.130%	58,401	3651	6.252%	1.878%	(1.581%, 2.176%)	1.117	(1.07, 1.167)	<0.0001
Heart failure	59,927	3140	5.240%	60,963	2426	3.979%	1.260%	(1.024%, 1.497%)	1.119	(1.062, 1.181)	<0.0001
Cardiac arrest	64,406	731	1.135%	64,486	563	0.873%	0.262%	(0.153%, 0.37%)	1.108	(0.992, 1.237)	0.0681
Acute myocardial infarction	61,651	2120	3.439%	62,076	1642	2.645%	0.794%	(0.602%, 0.99%)	1.11	(1.041, 1.184)	0.0015
Ischaemic stroke	62,731	1741	2.775%	62,774	1342	2.138%	0.638%	(0.466%, 0.809%)	1.101	(1.025, 1.183)	0.008
Haemorrhagic stroke	64,259	554	0.862%	64,239	453	0.705%	0.157%	(0.061%, 0.253%)	1.033	(0.912, 1.17)	0.6063
Pulmonary embolism	63,550	930	1.463%	63,659	747	1.173%	0.290%	(0.165%, 0.415%)	1.06	(0.962, 1.167)	0.2384
Cerebrovascular diseases	60,621	2684	4.428%	61,326	2177	3.550%	0.878%	(0.658%, 1.097%)	1.063	(1.005, 1.125)	0.034
Peripheral arterial disease	54,638	5415	9.911%	57,655	4514	7.829%	2.081%	(1.748%, 2.414%)	1.082	(1.04, 1.126)	<0.0001
Chronic ischaemic heart disease	56,281	4280	7.605%	57,445	3437	5.983%	1.622%	(1.329%, 1.914%)	1.096	(1.048, 1.146)	<0.0001
Thrombotic venous disease	62,202	2455	3.947%	64,140	1722	2.685%	1.262%	(1.064%, 1.46%)	1.26	(1.185, 1.341)	<0.0001
Conduction diseases	53,536	6031	11.27%	55,016	5363	9.748%	1.517%	(1.152%, 1.882%)	0.99	(0.954, 1.027)	0.5793
Valve disorders	58,901	3657	6.209%	59,952	2943	4.909%	1.300%	(1.039%, 1.56%)	1.08	(1.029, 1.134)	0.0018
Carditis	63,651	716	1.125%	64,057	532	0.831%	0.294%	(0.186%, 0.402%)	1.153	(1.03, 1.29)	0.0133
Rheumatic heart disease	62,566	1675	2.677%	62,923	1357	2.157%	0.521%	(0.351%, 0.691%)	1.057	(0.983, 1.135)	0.133

MACE, major adverse cardiovascular events, including myocardial infarction, stroke, cardiogenic shock, cardiac arrest and congestive heart failure. The Log-rank test was used to compare Kaplan–Meier curve.

### Acute cardiovascular events

Concise cardiovascular events were investigated in composite endpoints for acute myocardial infarction, ischaemic or haemorrhagic stroke and pulmonary embolism. The risk of acute myocardial infarction, including ST- and non-ST-elevation myocardial infarction, was slightly increased in patients with PN (HR 1.11; CI 1.041–1.184; p = 0.0015, Fig. 1b). The risks for stroke or pulmonary embolism were not different between patients with PN and propensity-matched controls.

### Chronic vasculopathic and cardiac diseases

We assessed the risks of vasculopathy after diagnosis of PN by investigating composite endpoints for diseases of the venous, coronary, peripheral arterial and cerebrovascular systems. Higher risks were found for severe thrombotic venous diseases including venous thrombosis and postthrombotic syndrome (HR 1.26; CI 1.185–1.341; p < 0.0001, see Fig. 1c), chronic ischaemic heart diseases including angina pectoris (HR 1.096; CI 1.048–1.146; p < 0.0001) and peripheral arterial diseases including atherosclerosis, arterial embolism, aortic aneurysm and gangrene (HR 1.082; CI 1.04–1.126; p < 0.0001).

For structural heart diseases, we investigated composite endpoints for rheumatic heart disease, carditis (including myo-, endo-, and pericarditis), conduction diseases and valve disorders. Only the latter showed higher risks in patients with PN and included non-rheumatic valve diseases as well as patient histories of valve replacement (HR 1.08; CI 1.029–1.134; p = 0.0018, see Fig. 1d).

### Sensitivity analysis

In order to ensure validity of the diagnosis of PN in the patient cohort, we recruited a cohort with an additional documented diagnosis of PN at least 1 month after the initial diagnosis, comprising a total of 21,529 patients. Assessing cardiovascular outcomes, we found similar results to the larger cohort of patients with only one registration of PN, however, due to lower case numbers, some results were statistically not significant (see Supplementary Fig. S2A–D). Most notably, we confirmed a higher risk of MACE (HR 1.162; CI 1.083–1.246; p < 0.0001), heart failure (HR 1.28; CI 1.174–1.395; p < 0.0001) and myocardial infarction (HR 1.164; CI 1.05–1.29; p = 0.0038).

### Ethnicity-specific sub-analysis

**Fig. 2:**
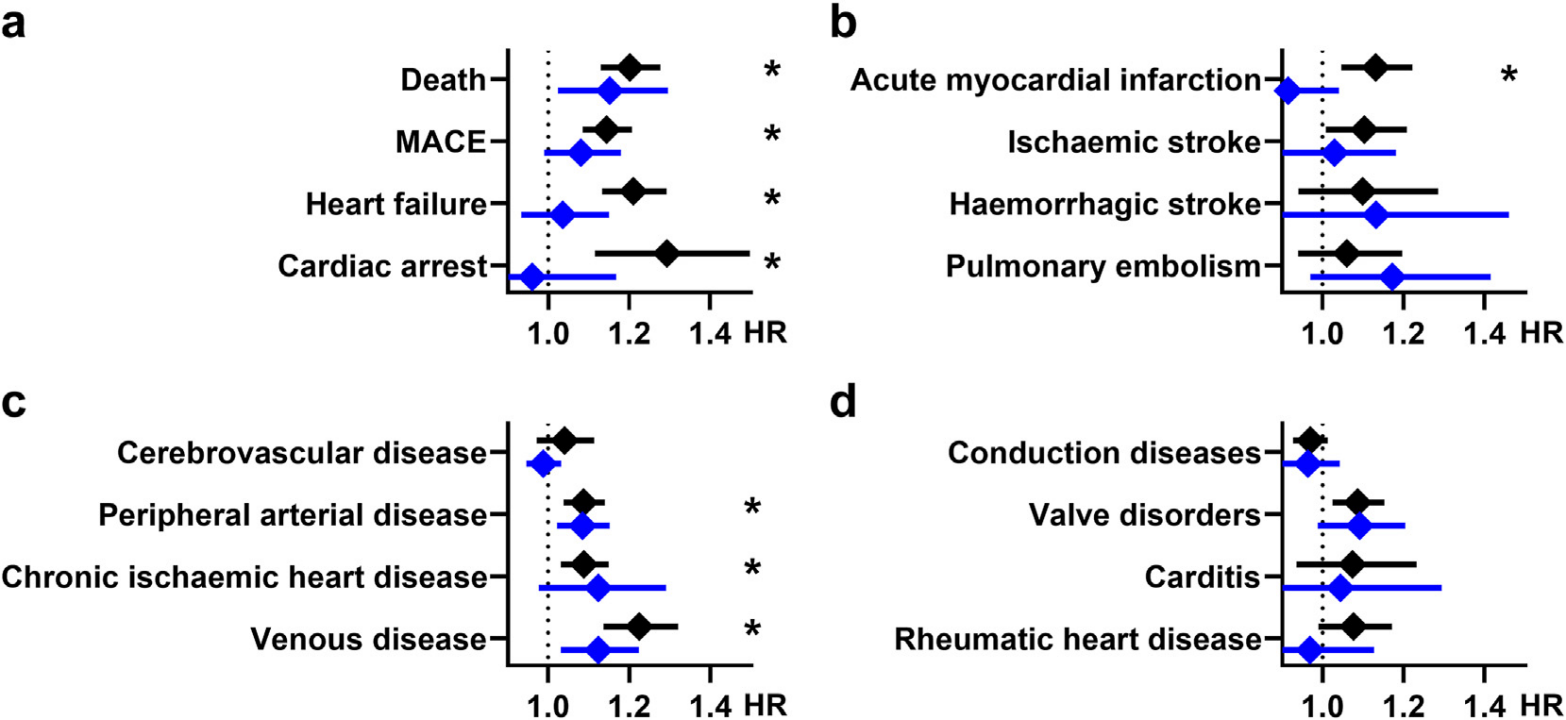
Hazard ratios (HR) for (a) mortality endpoints (MACE, major adverse cardiovascular events), (b) acute atheromatous events, (c) vascular diseases and (d) structural cardiac diseases are shown comparing either prurigo nodularis patients with skin of colour (blue symbols) or white (black symbols) patients with prurigo nodularis with matched controls. Bars show mean hazard ratios ±95%-confidence interval. ∗p < 0.003125 (Log-rank test). n = 13,612 per group.

As PN was shown in other studies^9^, ^10^, ^11^ to be more frequent in Black and African American individuals, we conducted an ethnicity-specific analysis to identify RD between ethnic backgrounds. To this end, sub-groups with control patients or patients with PN with skin of colour (n = 13,612 for both groups after propensity matching) and white control patients or patients with PN (n = 42,553) were identified. Here, less HCOs provided EHRs (n = 58 for patients with skin of colour and n = 65 for white patients) as this data is not collected stringently at all HCOs; hence, EHRs without demographical data including ethnicity were excluded. Overall, the data for white patients with PN matched the results obtained for the full cohort with PN, while the group of patients with PN with skin of colour showed no higher risks of major or acute cardiovascular events and also no higher mortality (see Fig. 2a–d).

### Sex-specific sub-analysis

**Fig. 3:**
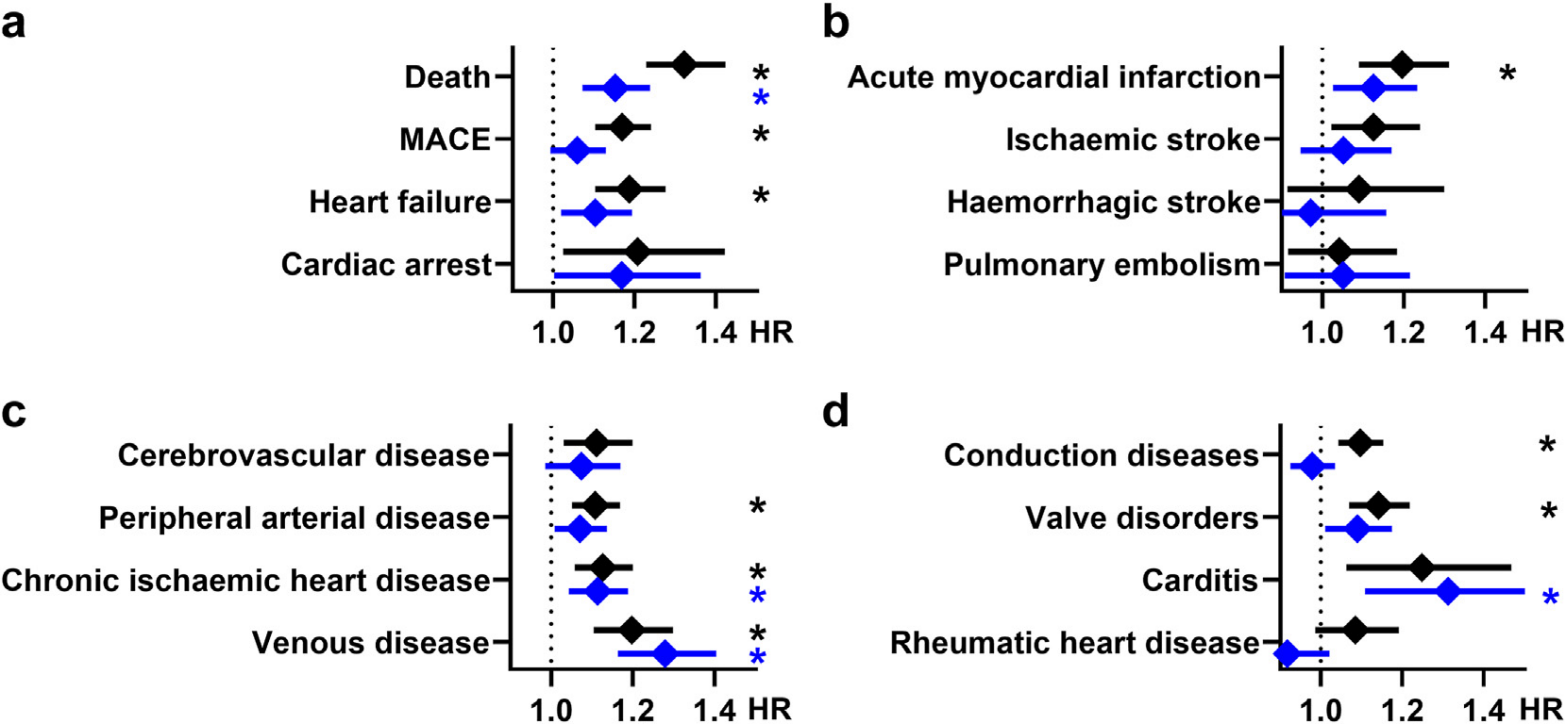
Hazard ratios (HR) for (a) mortality endpoints (MACE, major adverse cardiovascular events), (b) acute atheromatous events, (c) vascular diseases and (d) structural cardiac diseases are shown comparing either male (blue symbols) or female (black symbols patients with prurigo nodularis with matched controls. Bars show mean hazard ratios ±95%-confidence interval. ∗p < 0.003125 (Log-rank test). n = 38,250 per group.

In a sex-specific analysis, we stratified the patient and control group into sub-groups with only female (n = 38,250) or male patients (n = 26,427). Overall, the hazard ratios in the female patients with PN were similar to the non-stratified cohort with PN, while in males no increased risks of major or acute cardiovascular events were observed (see Fig. 3a–d). However, male patients with PN had an increased risk of chronic ischaemic heart disease (HR 1.113; CI 1.043–1.188; p = 0.0013) and complicated venous disease (HR 1.278; CI 1.163–1.404; p < 0.0001) en par with female patients (HR 1.126; 1.057–1.2; p = 0.0002; and HR 1.197; CI 1.104–1.298; p < 0.0001), in addition, a markedly increased risk of carditis (HR 1.312; CI 1.108–1.552; p = 0.0015) was observed in male but not in female patients.

### Impact of dupilumab treatment on the investigated outcomes

**Fig. 4:**
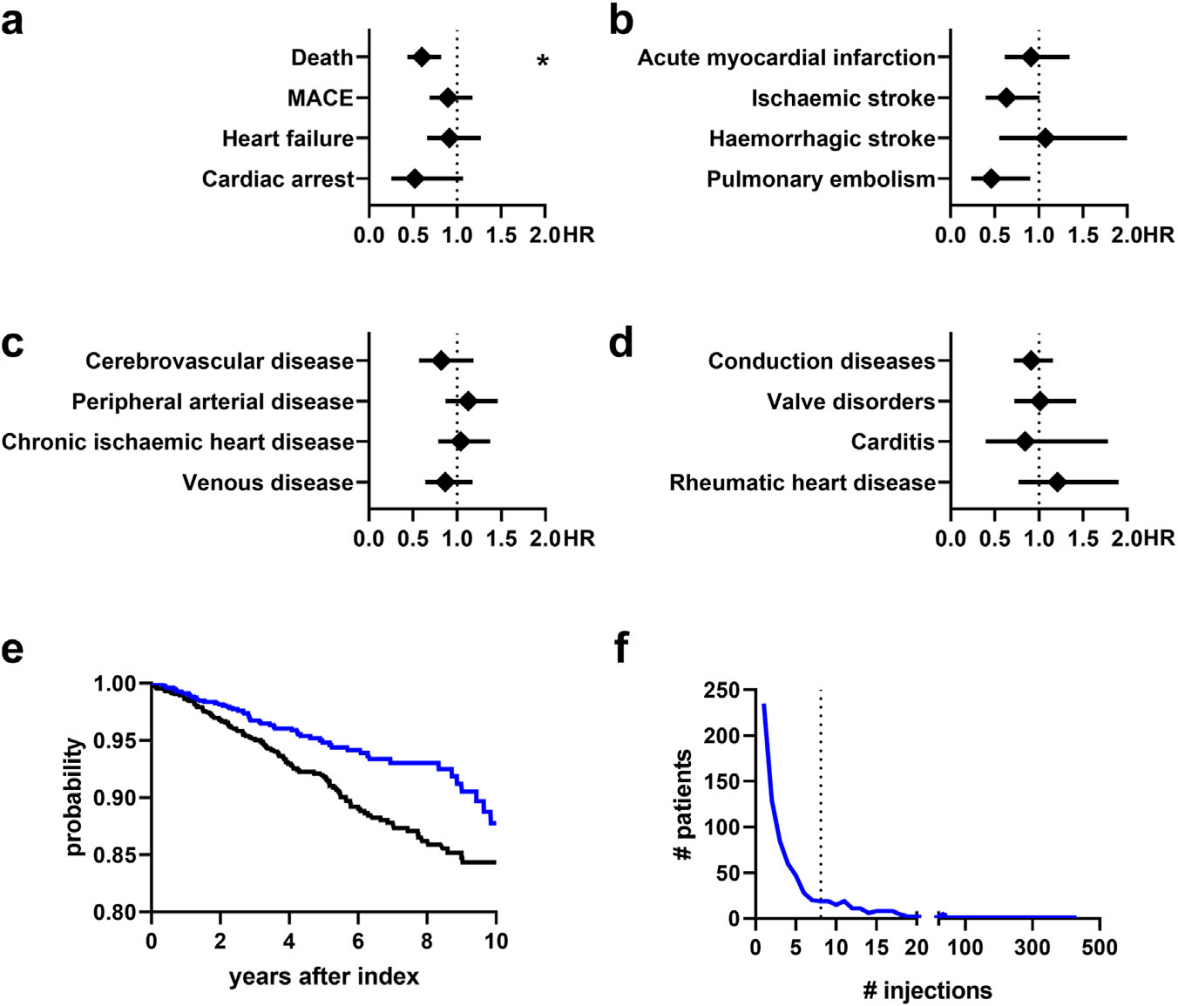
Hazard ratios (HR) for (a) mortality endpoints (MACE, major adverse cardiovascular events), (b) acute atheromatous events, (c) vascular diseases and (d) structural cardiac diseases are shown comparing patients with prurigo nodularis treated with standard therapies but not dupilumab with patients treated with dupilumab. Bars show mean hazard ratios ±95%-confidence interval. ∗p < 0.003125 (Log-rank test). (e) Kaplan-Meyer curves showing survival probabilities regarding mortality over time for patients with prurigo nodularis treated with dupilumab (blue) or only with other approved medications (black, see Supplementary Table S1 for a list of included drugs). (f) Frequency of the number of dupilumab injections registered in the dupilumab-treated group; dotted line shows mean value (8.086), median: 3 injections. n = 1454 per group.

To investigate the effect of dupilumab on cardiovascular risks in patients with PN, we recruited a cohort of patients with PN treated with dupilumab and a control cohort treated with at least one other approved medication in the absence of dupilumab. We included 1454 patients treated with dupilumab. Notably, we found that other approved standard medications were used more frequently in patients also treated with dupilumab (see Supplementary Table S2 for baseline characteristics). This could indicate a higher disease severity, but could also show better access to health care and possibly a higher socioeconomic status of patients treated with dupilumab. To account for this, we extended the propensity-matching strategy in this analysis and included a list of additional medications (Supplementary Table S1); the groups after matching had no significant differences in the administration of these drugs. Finally, we found that the all-cause mortality was significantly lower with dupilumab (HR 0.599, CI 0.436–0.882; p = 0.0013, Fig. 4a and e). However, the low case numbers did not suffice to demonstrate statistically significant lower cardiovascular risks (see Fig. 4a–d). The mean number of dupilumab injections was 8.086 (median: 3, Fig. 4f) indicating sufficient therapy adherence.

## Discussion

Chronic inflammatory diseases impose a high medical burden and cardiovascular sequelae are well-described in particular. One may assume that the underlying chronic inflammation is a common denominator of the risk for cardiovascular disease in multiple chronic inflammatory diseases, including prurigo nodularis.^34^ Regarding chronic skin diseases, markedly increased cardiovascular risks were found for primarily Th1- and Th17-driven inflammation, for example, in patients with cutaneous lupus erythematosus^28^ or psoriasis.^35^ In addition, some studies indicated protective cardiovascular effects of treatment with antipsoriatic biologicals showing possible mechanistic overlaps of skin inflammation and vascular damage.^36^ In contrast, current data on cardiovascular comorbidities in patients with PN is limited but smaller studies showed higher risks of hypertension, ischaemic heart disease and more frequent occurrence of traditional cardiovascular risk factors.^11^, ^12^, ^13^ In line, other inflammatory skin diseases with similar Th2-related pathophysiology showed a weak association with higher cardiovascular risks, including AD and allergic asthma.^37^ Nevertheless, a relation between Th2-mediated diseases and cardiovascular comorbidities and a pathogenetic link remains disputed.

Our study provides new insights into cardiovascular risks in patients with PN by analysing a much larger cohort than previous studies that allowed for stratification and sub-analyses with regard to demographics and treatments. First, we demonstrated that the global patient cohort with PN at baseline showed higher frequencies of known cardiovascular risk factors including nicotine dependence, obesity, hypertension, and diabetes mellitus as well as neoplasms and neuropsychiatric diseases. This is in line with findings of other studies.^11^, ^12^, ^13^ We then employed a stringent propensity-score matching strategy in order to analyse cardiovascular risks in patients with PN independently of pre-existing cardiovascular risk factors and major comorbidities. We showed higher mortality and higher risks of major adverse cardiovascular outcomes as well as a range of chronic vascular diseases including chronic ischaemic heart disease, peripheral arterial disease and thrombotic venous disease (Fig. 1). A sensitivity analysis was conducted in a cohort of patients with PN that had two documented diagnoses of PN with at least one month difference, as the accuracy of ICD-10 codes was shown to increase with multiple registrations of the same code for other diseases.^38^ Here, despite lower case numbers, we confirmed higher cardiovascular risks in patients with PN (Supplementary Fig. S2A–D).

A sub-analysis of patients with PN with skin of colour showed lower cardiovascular risks than in white patients with PN despite higher frequency of PN in people with skin of colour (Fig. 2). This is in contrast to results of other studies that previously showed higher mortality and cardiovascular risks in people with skin of colour but did not employ adjustment for other known risk factors such as diabetes mellitus or smoking.^13^ In a sex-specific sub-analysis we showed that the observed cardiovascular risks mainly affected female patients with PN (Fig. 3).

Regarding a potential pathogenetic link between cardiovascular diseases and PN, in line with other Th2-related diseases, no precise mechanism has been identified, yet. Strikingly, we found that patients with PN treated with dupilumab had lower mortality compared to standard treatment regimens (Fig. 4), however, this effect was relatively weak. The lack of differences in cardiovascular risks might be due to the relatively low sample sizes. A possible explanation for better survival after blocking interleukin (IL)-4 and IL-13 might simply be reduced psychological and physical distress. Other studies indicated that Th2-associated mediators could be directly involved in the pathogenesis of arteriosclerosis, e.g., by activation and recruitment of eosinophils into arteriosclerotic plaques. In line, dupilumab was shown to modulate pro-arteriosclerotic genes in patients with AD.^39^ Dupilumab could thus play a role in the risk reduction of major sequelae in patients with PN. Further, transcriptomic analyses of patients with PN showed increased activation of the renin-angiotensin-aldosterone system (RAAS), possibly driving vascular inflammation and cardiac remodelling in patients with PN.^40^ Single-cell RNA sequencing of PN skin lesions further showed a dysregulated fibroblast activity,^41^ leading to an increased mesenchymal remodelling possibly causing structural changes in the cardiac tissue. Another shared risk factor might be Helicobacter (H.) pylori infection that is associated with cardiovascular disease^42^^,^^43^ that is more frequent in patients with PN.^43^

Our findings have important clinical implications for the management and primary prophylaxis in patients with PN. Although no novel PN-specific treatment rationale can be derived from the presented data, the potential risk of subsequent cardiovascular disease should be considered in the care of patients with PN, which includes screening and optimal management of other additional cardiovascular risk factors.

This study has several limitations to be appreciated. First, due to the retrospective observational design of the study, differences in cardiovascular risks found here cannot be causally attributed to underlying PN and reverse causality might play a role. Also, due to the observational design, confounding of the data by a survivorship bias is possible despite relatively low mortality and long follow-up periods reported here. Although an extensive matching model was employed, measured and unmeasured confounders could have biased the data. This is especially important for the analysis of dupilumab effects since the cohort size is relatively small and the median measured duration of treatment was short (median: 3 injections); thus, multiple unadjusted confounding factors might have gravely influenced the results. Second, limitations arise due to the design of the TriNetX database: The database provides digital EHRs and is thus susceptible to misdiagnosis or false coding of diagnoses and demographic information. Especially, the classification of cardiovascular risk factors used for propensity-matching might be reflected incorrectly. Also, for the ethnicity-specific sub-analysis, not all HCOs included in the global analysis could be used due to the lack of demographic data collection in some regions; particularly, only 52–71 out of 103 HCOs provided EHRs on cohorts of people with skin of colour. This implies a bias due to the HCO distribution; HCO effects and clustering cannot be controlled in this study due to the level of anonymisation of the data. In the analysis of dupilumab-treatment effects, co-existing or miscoded atopic dermatitis or other approved indications might be confounding the data, and exclusion of patients with atopic dermatitis yielded cohort sizes too low for analysis. Further, the diagnosis of PN as well as the analysed outcomes were not validated by additional recorded parameters in the EHRs due to the lack of specific data from laboratory tests, further clinical examination or histology; however, in a sensitivity analysis with better validated diagnosis by requiring two registrations of the diagnosis of PN with at least 1 month difference showed similar results. Also, disease scores or data on severity and progression are not provided. Moreover, as patient records are reported by healthcare providers, accessibility of healthcare services is a potential confounder on a global scale. Finally, reported RD must be interpreted cautiously if case numbers are low for the outcome, moreover, multiplicity or circularity in the datasets while analysing multiple outcomes could not be excluded.

To conclude, we evaluated cardiovascular risks in patients with PN. By use of the large database of TriNetX, sample sizes were sufficient for stratification by ethnicity and sex. Traditional cardiovascular risk factors as well as malignant and neuropsychiatric diseases were more frequent in patients with PN, in line with other studies, which substantiates the validity of our findings. By employing a propensity-score matching strategy, we identified PN as an independent cardiovascular risk factor in white and female patients, but not so much in males and people with skin of colour. This is despite significantly higher cardiovascular risks found by others especially in people with skin of colour that might thus be due to additional confounding factors. However, it must be noted that the risk enhancement was significant but overall relatively weak. Additionally, we found slightly lower overall mortality in patients with PN that were treated with dupilumab; this has yet to be confirmed in prospective studies.

## Contributors

HO and RJL conceptualised the study. CDS, DT and RJL acquired funding. HO, KK, GH, HZ, RJL and KB analysed the data. HO and RJL verified the results. HO drafted the manuscript. KK, GH, HZ, CDS, PT, DT, RJL, and KB revised the manuscript. All authors read and approved the submitted version.

## Data sharing statement

The original contributions presented in the study are included in the article and the supplemental material, further inquiries can be directed to the corresponding author.

## Declaration of interests

The authors declare that this study was designed and conducted in the absence of any financial or commercial relationships that could be construed as a potential conflict of interest. HZ received travel honoraria and support for meeting attendance from TriNetX, Pfizer, UCB Pharma, Almirall and Janssen unrelated to the current work.

## Figures and Tables

**Supplementary Fig. S2:**