# Supplementary material for: Increased cardiovascular risks and mortality in prurigo nodularis: a global cohort study
Source: eBioMedicine. 2024 Apr 16;103:105123. doi: 10.1016/j.ebiom.2024.105123 (PMC11035034; doi:10.1016/j.ebiom.2024.105123)
Supplement: Supplementary Tables [file mmc1.docx]

# Supplemental Tables

| **drug** | **RxNorm** |
| --- | --- |
| prednisone | 8640 |
| prednisolone | 8638 |
| methylprednisolone | 6902 |
| dexamethasone | 3264 |
| clobetasol | 2590 |
| mometasone | 108118 |
| mycophenolate | 68149 |
| mycophenolic | 7145 |
| ciclosporine | 3008 |
| azathioprine | 1256 |
| cetirizine | 20610 |
| desloratadine | 275635 |
| loratadine | 28889 |
| fexofenadine | 87636 |
| capsaicin | 1992 |
| naloxone | 7242 |
| naltrexone | 7243 |
| gabapentin | 25489 |
| pregabalin | 187832 |
| doxepin | 3638 |
| amitriptyline | 704 |
| fluoxetine | 4493 |
| sertraline | 36437 |
| paroxetine | 32937 |
| mirtazapine | 15996 |

**Suppl. Tab. 1.** List of alternative approved medications to treat prurigo nodularis including corticosteroids, immunosuppressants and –modulators, antihistamines, opioid-receptor antagonists, anticonvulsants and antidepressants. Usage of at least one of the listed drugs was required for inclusion to the non-dupilumab treated control group of patients with PN; all drugs listed were included in the propensity-score matching model.

|  |  | **Before matching:** | | | | | | **After matching:** | | | | | |
| --- | --- | --- | --- | --- | --- | --- | --- | --- | --- | --- | --- | --- | --- |
| **Characteristic:** |  | **Dupilumab-treated** | | **non-Dupilumab-treated** | | **p-value** | **SMD** | **Dupilumab-treated** | | **non-Dupilumab-treated** | | **p-value** | **SMD** |
| Age at Index (years, ± SD) | | 53.41 | 16.51 | 55.26 | 14.89 | < 0.0001 | 0.118 | 53.47 | 16.50 | 52.26 | 15.47 | 0.043 | 0.075 |
|  | ICD-10 | n = | % | n = | % |  |  | n = | % | n = | % |  |  |
| Total |  | 1461 | 100.0% | 52330 | 100.0% |  |  | 1454 | 100.0% | 1454 | 100.0% |  |  |
| Not Hispanic or Latino |  | 1085 | 74.3% | 37259 | 71.2% | 0.011 | 0.069 | 1079 | 74.2% | 1103 | 75.9% | 0.304 | 0.038 |
| Female |  | 850 | 58.2% | 30380 | 58.1% | 0.924 | 0.003 | 848 | 58.3% | 856 | 58.9% | 0.763 | 0.011 |
| White |  | 681 | 46.6% | 32154 | 61.4% | < 0.0001 | 0.301 | 678 | 46.6% | 656 | 45.1% | 0.413 | 0.030 |
| Neoplasms | C00-D49 | 733 | 50.2% | 31904 | 61.0% | < 0.0001 | 0.219 | 733 | 50.4% | 706 | 48.6% | 0.317 | 0.037 |
| Essential (primary) hypertension | I10 | 677 | 46.3% | 25605 | 48.9% | 0.051 | 0.052 | 676 | 46.5% | 639 | 43.9% | 0.168 | 0.051 |
| Disorders of lipoprotein metabolism and other lipidemias | E78 | 540 | 37.0% | 24908 | 47.6% | < 0.0001 | 0.217 | 540 | 37.1% | 509 | 35.0% | 0.231 | 0.044 |
| Chronic lower respiratory diseases | J40-J4A | 521 | 35.7% | 15993 | 30.6% | < 0.0001 | 0.109 | 519 | 35.7% | 509 | 35.0% | 0.698 | 0.014 |
| Overweight, obesity and other hyperalimentation | E65-E68 | 405 | 27.7% | 15922 | 30.4% | 0.027 | 0.060 | 405 | 27.9% | 406 | 27.9% | 0.967 | 0.002 |
| Diabetes mellitus | E08-E13 | 315 | 21.6% | 12992 | 24.8% | 0.004 | 0.077 | 315 | 21.7% | 299 | 20.6% | 0.467 | 0.027 |
| Nicotine dependence | F17 | 262 | 17.9% | 9464 | 18.1% | 0.881 | 0.004 | 262 | 18.0% | 239 | 16.4% | 0.259 | 0.042 |
| Chronic kidney disease (CKD) | N18 | 155 | 10.6% | 5768 | 11.0% | 0.619 | 0.013 | 155 | 10.7% | 131 | 9.0% | 0.135 | 0.055 |
| Family history of ischaemic heart disease and other diseases of the circulatory system | Z82.4 | 86 | 5.9% | 2942 | 5.6% | 0.665 | 0.011 | 86 | 5.9% | 75 | 5.2% | 0.372 | 0.033 |
| Clobetasol |  | 819 | 56.1% | 16067 | 30.7% | < 0.0001 | 0.529 | 812 | 55.8% | 778 | 53.5% | 0.205 | 0.047 |
| Prednisone |  | 711 | 48.7% | 15731 | 30.1% | < 0.0001 | 0.388 | 706 | 48.6% | 710 | 48.8% | 0.882 | 0.006 |
| Gabapentin |  | 495 | 33.9% | 12919 | 24.7% | < 0.0001 | 0.203 | 491 | 33.8% | 492 | 33.8% | 0.969 | 0.001 |
| Cetirizine |  | 420 | 28.7% | 8468 | 16.2% | < 0.0001 | 0.305 | 417 | 28.7% | 421 | 29.0% | 0.870 | 0.006 |
| Methylprednisolone |  | 401 | 27.4% | 13051 | 24.9% | 0.029 | 0.057 | 398 | 27.4% | 386 | 26.5% | 0.616 | 0.019 |
| Dexamethasone |  | 390 | 26.7% | 12793 | 24.4% | 0.049 | 0.052 | 389 | 26.8% | 363 | 25.0% | 0.271 | 0.041 |
| Doxepin |  | 235 | 16.1% | 2338 | 4.5% | < 0.0001 | 0.390 | 230 | 15.8% | 218 | 15.0% | 0.538 | 0.023 |
| Loratadine |  | 225 | 15.4% | 7438 | 14.2% | 0.201 | 0.033 | 223 | 15.3% | 233 | 16.0% | 0.610 | 0.019 |
| Mometasone |  | 213 | 14.6% | 5503 | 10.5% | < 0.0001 | 0.123 | 211 | 14.5% | 211 | 14.5% | 1.000 | < 0.0001 |
| Fexofenadine |  | 166 | 11.4% | 4391 | 8.4% | < 0.0001 | 0.100 | 164 | 11.3% | 165 | 11.3% | 0.953 | 0.002 |
| Naloxone |  | 164 | 11.2% | 5250 | 10.0% | 0.135 | 0.039 | 164 | 11.3% | 151 | 10.4% | 0.438 | 0.029 |
| Sertraline |  | 156 | 10.7% | 6320 | 12.1% | 0.105 | 0.044 | 156 | 10.7% | 151 | 10.4% | 0.763 | 0.011 |
| Prednisolone |  | 125 | 8.6% | 3746 | 7.2% | 0.041 | 0.052 | 124 | 8.5% | 123 | 8.5% | 0.947 | 0.002 |
| Ciclosporine |  | 128 | 8.8% | 1161 | 2.2% | < 0.0001 | 0.290 | 122 | 8.4% | 111 | 7.6% | 0.452 | 0.028 |
| Mycophenolate mofetil |  | 125 | 8.6% | 1172 | 2.2% | < 0.0001 | 0.282 | 120 | 8.3% | 111 | 7.6% | 0.537 | 0.023 |
| Pregabalin |  | 114 | 7.8% | 3394 | 6.5% | 0.044 | 0.051 | 114 | 7.8% | 108 | 7.4% | 0.675 | 0.016 |
| Amitriptyline |  | 113 | 7.7% | 4472 | 8.5% | 0.273 | 0.030 | 113 | 7.8% | 126 | 8.7% | 0.380 | 0.033 |
| Fluoxetine |  | 103 | 7.1% | 4248 | 8.1% | 0.140 | 0.040 | 103 | 7.1% | 105 | 7.2% | 0.886 | 0.005 |
| Mirtazapine |  | 89 | 6.1% | 2300 | 4.4% | 0.002 | 0.076 | 89 | 6.1% | 92 | 6.3% | 0.818 | 0.009 |
| Naltrexone |  | 58 | 4.0% | 560 | 1.1% | < 0.0001 | 0.186 | 56 | 3.9% | 60 | 4.1% | 0.705 | 0.014 |
| Paroxetine |  | 47 | 3.2% | 2667 | 5.1% | 0.001 | 0.094 | 47 | 3.2% | 43 | 3.0% | 0.668 | 0.016 |
| Azathioprine |  | 35 | 2.4% | 737 | 1.4% | 0.002 | 0.072 | 35 | 2.4% | 31 | 2.1% | 0.618 | 0.018 |
| Capsaicin |  | 28 | 1.9% | 1185 | 2.3% | 0.377 | 0.024 | 28 | 1.9% | 27 | 1.9% | 0.892 | 0.005 |
| Mycophenolic acid |  | 18 | 1.2% | 503 | 1.0% | 0.297 | 0.026 | 18 | 1.2% | 17 | 1.2% | 0.865 | 0.006 |
| Desloratadine |  | 16 | 1.1% | 737 | 1.4% | 0.315 | 0.028 | 16 | 1.1% | 11 | 0.8% | 0.334 | 0.036 |

**Suppl. Tab. 2.** Baseline characteristics before and after propensity-score matching for the Dupilumab- and non-Dupilumab treated groups. SD, standard deviation; SMD, standardised mean difference; uncorrected p-values are shown (t-test). All covariates shown here were included in the propensity-score matching model.
